# 11p11.12p12 duplication in a family with intellectual disability and craniofacial anomalies

**DOI:** 10.1186/s12920-021-00945-8

**Published:** 2021-04-09

**Authors:** Xuejiao Chen, Huihui Xu, Weiwu Shi, Feng Wang, Fenfen Xu, Yang Zhang, Jun Gan, Xiong Tian, Baojun Chen, Meizhen Dai

**Affiliations:** 1 Medical Research Center, Taizhou Hospital of Zhejiang Province Affiliated to Wenzhou Medical University, Linhai, Zhejiang China; 2 Department of Neurology, Taizhou Hospital of Zhejiang Province Affiliated to Wenzhou Medical University, Linhai, Zhejiang China; 3 Department of Pharmacy, Taizhou Hospital of Zhejiang Province Affiliated to Wenzhou Medical University, Linhai, Zhejiang China; 4 Department of Public Research Platform, Taizhou Hospital of Zhejiang Province Affiliated to Wenzhou Medical University, Linhai, Zhejiang China; 5 Department of Mental Health, Taizhou Hospital of Zhejiang Province Affiliated to Wenzhou Medical University, Linhai, Zhejiang China

**Keywords:** 11p11.12p12 duplication, Intellectual disability, Chromosome 11, Molecular cytogenetics, Genetic counselling

## Abstract

**Background:**

Potocki–Shaffer syndrome (PSS) is a rare contiguous gene deletion syndrome marked by haploinsufficiency of genes in chromosomal region 11p11.2p12. Approximately 50 cases of PSS have been reported; however, a syndrome with a PSS-like clinical phenotype caused by 11p11.12p12 duplication has not yet been reported.

**Methods:**

11p11.12p12 duplication syndrome was identified and evaluated using a multidisciplinary protocol. Diagnostic studies included intelligence testing, thorough physical examination, electroencephalography (EEG), magnetic resonance imaging (MRI) of the brain, ultrasonography, biochemical tests and karyotype analysis. Next-generation sequencing analysis clarified the location of the chromosomal variations, which was confirmed by chromosome microarray analysis (CMA). Whole-exome sequencing (WES) was performed to exclude single nucleotide variations (SNVs). A wider literature search was performed to evaluate the correlation between the genes contained in the chromosomal region and clinical phenotypes.

**Results:**

The proband was a 36-year-old mother with intellectual disability (ID) and craniofacial anomalies (CFA). She and her older son, who had a similar clinical phenotype, both carried the same 11p11.12p12 duplication with a copy number increase of approximately 10.5 Mb (chr11:40231033_50762504, GRCh37/hg19) in chromosome bands 11p11.12p12. In addition, she gave birth to a child with a normal phenotype who did not carry the 11p11.12p12 duplication. By literature research and DECIPHER, we identified some shared and some distinct features between this duplication syndrome and PSS. One or more of *ALX4, SLC35C1*, *PHF21A* and *MAPK8IP1* may be responsible for 11p11.12p12 duplication syndrome.

**Conclusions:**

We present the first report of 11p11.12p12 duplication syndrome. It is an interesting case worth reporting. The identification of clinical phenotypes will facilitate genetic counselling. A molecular cytogenetic approach was helpful in identifying the genetic aetiology of the patients and potential candidate genes with triplosensitive effects involved in 11p11.12p12 duplication.

## Background

With the increasing clinical application of molecular cytogenetics, such as next-generation sequencing analysis and chromosome microarray analysis (CMA), increasing numbers of chromosomal deletions/microdeletions and duplications/microduplications have been detected and identified, affording new development opportunities for genetics and new challenges for clinical genetic counselling, especially prenatal genetic counselling. Potocki–Shaffer syndrome (PSS) is caused by a rare contiguous gene deletion in chromosomal region 11p11.2p12 (chr11: 43421550_48821552, GRCh37/hg19). Our patient’s clinical phenotype is also similar but not entirely identical to that of PSS; however, a syndrome caused by 11p11.12p12 duplication has not yet been reported. Here, we report a de novo 11p11.12p12 duplication in a small family presenting with intellectual disability (ID) and craniofacial anomalies (CFA). The duplication encompasses approximately 10 Mb (chr11: 40231033_50762504, GRCh37/hg19), which cannot be distinguished by G-banding 400-band resolution in karyotype analysis; therefore, it may also be called a microduplication.

The most common phenotype caused by chromosomal abnormalities is ID, which affects approximately 1% of the population [1]. There are approximately 1900 high-/moderate-confidence ID-causing genes that are annotated in the Genomics England ID panel and DDG2P (https://www.ebi.ac.uk/gene2phenotype). The gene-disease identities of 484 genes (including many non-ID genes) have been curated by ClinGen (https://www.clinicalgenome.org/) [2]. The organ specificity of four genes involved in the 11p11.12p12 chromosomal region, namely, *SLC35C1*, *PEX16*, *PHF21A*, and *RAPSN*, is considered in DDG2P to be related to brain/cognition, and the patients’ phenotypes suggest that one or more genes that likely contribute to the clinical phenotype in this syndrome are located in the duplicated region.

## Methods

### Karyotype analysis

Karyotype analysis was performed in 6 main members of the family to identify the genetic aetiology. Chromosomes were obtained from cultured peripheral lymphocytes. The conventional technique of G banding analysis was used. Twenty-five metaphases of the family members were analysed at 550 chromosome band resolution. The International System for Human Cytogenomic Nomenclature (ISCN, 2016) served as the reference for describing the chromosomes.

### Next-generation sequencing analysis

To clarify the location of the chromosomal variations and exclude normal chromosomal variations, 11 members of the family underwent next-generation sequencing analysis, which was also performed in 7270 pregnant women to detect chromosomal microdeletions and microduplications for prenatal diagnosis. DNA was extracted from patient whole blood or amniotic fluid using a MagPure Universal DNA LQ Kit. A DNA library was obtained after the DNA was fragmented, end-repaired, linker-ligated and PCR-amplified. Then, the library DNA was sequenced using a BioelectronSeq 4000 instrument. The kits used in this study included the Ion Plus Fragment Library Kit, the Ion PI Hi-Q OT2 200 Templating Kit, the Ion PI Hi-Q 200 Sequence Kit and the Ion PI Chip V3. All reagents were provided by Dongguan BoaoMuhua Gene Technology Co, Ltd, China.

### Chromosome microarray analysis

CMA was used to confirm the location of the chromosomal variations in the family and detect chromosomal microdeletions and microduplications in 2906 pregnant women for prenatal diagnosis. Human genomic DNA was extracted from whole blood or amniotic fluid using a QIAamp DNA Mini Kit. The following standard experimental procedures were performed: digestion, ligation, polymerase chain reaction (PCR), PCR purification, fragmentation, labelling, hybridization, washing, staining, and scanning. The instrument was an Affymetrix GeneChip System 3000D x v.2 chip system. The CytoScan 750 K Array includes 200,000 genotype-able SNPs and 550,000 non-polymorphic probes. All genotyping and copy number analysis were performed with Chromosome Analysis Suite (ChAS) Software version 3.0. All data were required to pass quality control (QC) metrics, including the median of the absolute values of all pairwise differences ≤ 0.25, SNPQC ≥ 15, and a waviness standard deviation ≤ 0.12.

### Whole-exome sequencing (high-throughput testing)

Whole-exome sequencing (WES) was performed to exclude single nucleotide variations (SNVs). Genomic DNA was extracted using a QIAamp DNA Extraction Kit. The extracted DNA was fragmented with DNase and purified by magnetic beads, followed by PCR amplification and ligation of the adaptor sequence, which was twice captured and purified by a TruSight One Sequencing Panel (Illumina Inc, USA) and then amplified by PCR. The final library obtained after purification was sequenced in the exon regions of 4811 clinically relevant genes in a MiSeq sequencer (Illumina Inc, USA). The TruSight One Sequencing Panel is based on the Human Gene Mutation Database (HGMD Professional), the Online Mendelian Inheritance in Man (OMIM), the GeneTests website (www.genetests.org), and others from Illumina. Information about the commercial kits that ultimately incorporate genes relevant to diabetes-related gene sequencing is provided by the manufacturer (http://www.illumina.com/products/trusight-one-sequencing-panel.ilmn).

All the data were compared to the reference sequence (UCSC hg19) using the Burrows-Wheeler Aligner (BWA; http://bio-bwa.sourceforge.net/) algorithm. In the screening process, the clinical data were combined with bioinformatics software (PolyPhen2, LRT, Mutation Taster, etc.) to predict the results, function, variation and genetic model of each gene; these results were analysed to identify suspicious candidate mutations, which were verified by Sanger sequencing. PCR primers were designed for the sites of suspected candidate mutations. The corresponding sites of the parents’ genomes were detected.

## Results

### Clinical features

**Fig. 1:**
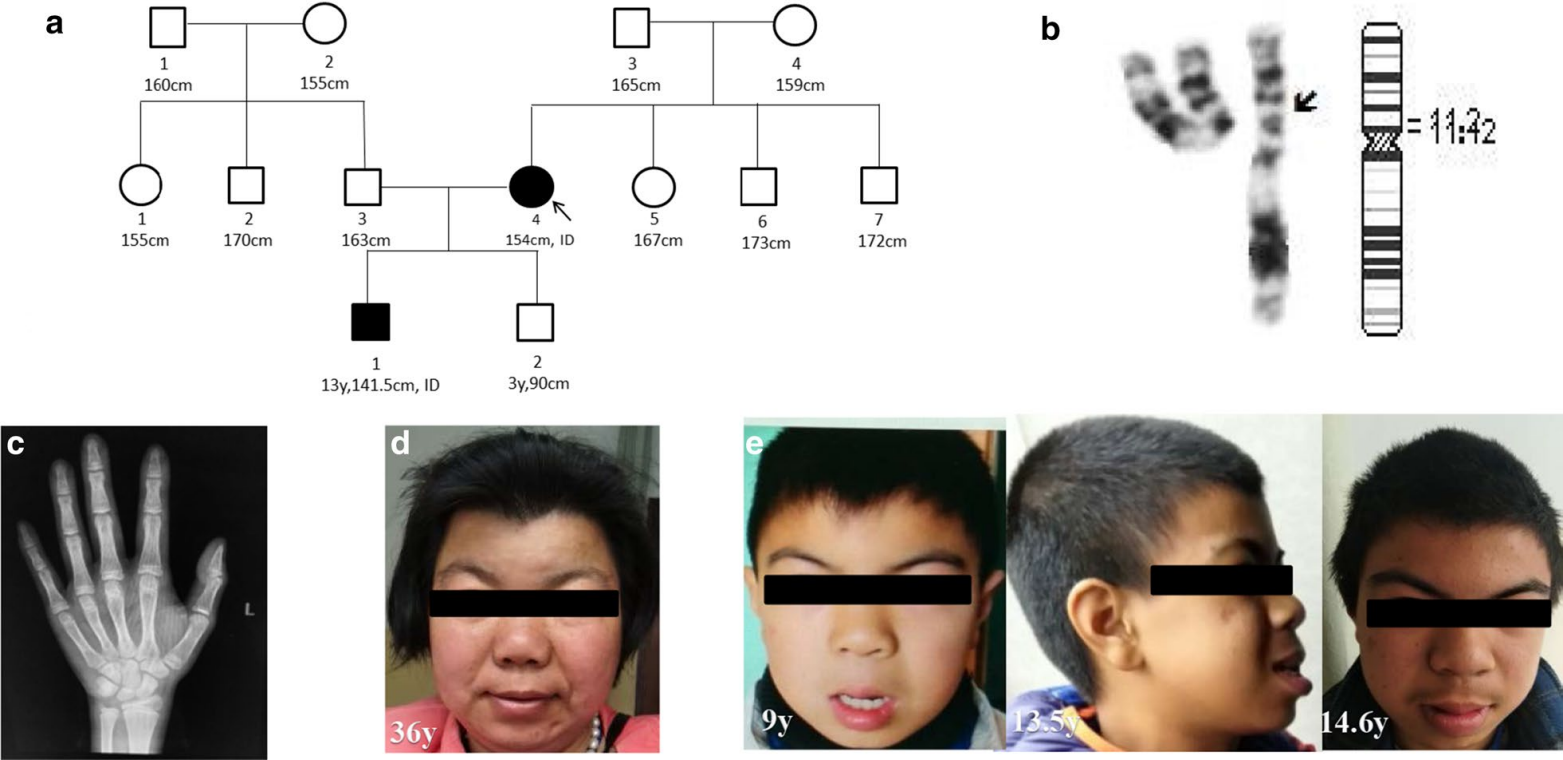
Clinical and genetic findings. **a** A pedigree of the family. Mother (II4), father (II3), older son (III1), younger son (III2). The mother (II4), who was the proband, had mild ID and was 154 cm (6 cm shorter than the mean height (160 cm) of the female family members) at 31 years of age. III1 had mild ID and was 141.5 cm tall (− 2.83 SD) at 13.5 years of age. III2 had normal intelligence and was 91 cm tall (− 1.53 SDs) at 3 years of age. **b** The partial karyotype and corresponding idiogram of chromosome 11 at the levels of the 550 bands. **c** III1’s bone age as determined by left wrist X-ray was 13.5 years, matching his actual age. d. Craniofacial features of II4, including low anterior hairline, hypertelorism, depressed nasal bridge, long philtrum, and slightly upturned corners of mouth. e. Craniofacial anomalies of III1, including low anterior hairline, thick eyebrows, long eyelashes, hypertelorism, long philtrum, risus sardonicus, upturned corners of mouth, thick lower lip vermilion, and carious teeth

The proband (II4, Fig. 1a) was a 31-year-old pregnant woman. She was 154 cm in height (− 1.22 SDs), shorter than the mean height (160 cm) of the women in the family by 6 cm. She showed mild ID, occasional dizziness, resting tremor, and CFA (Fig. 1d). Her Wechsler Adult Intelligence Scale (WAIS) test score was IQ = 56. She could take care of herself but could not work normally while chatting with others. Her language expression exhibited some difficulties; for example, she could not accurately read the number 888. She also had difficulty adding numbers from 1 to 10.

**Fig. 2:**
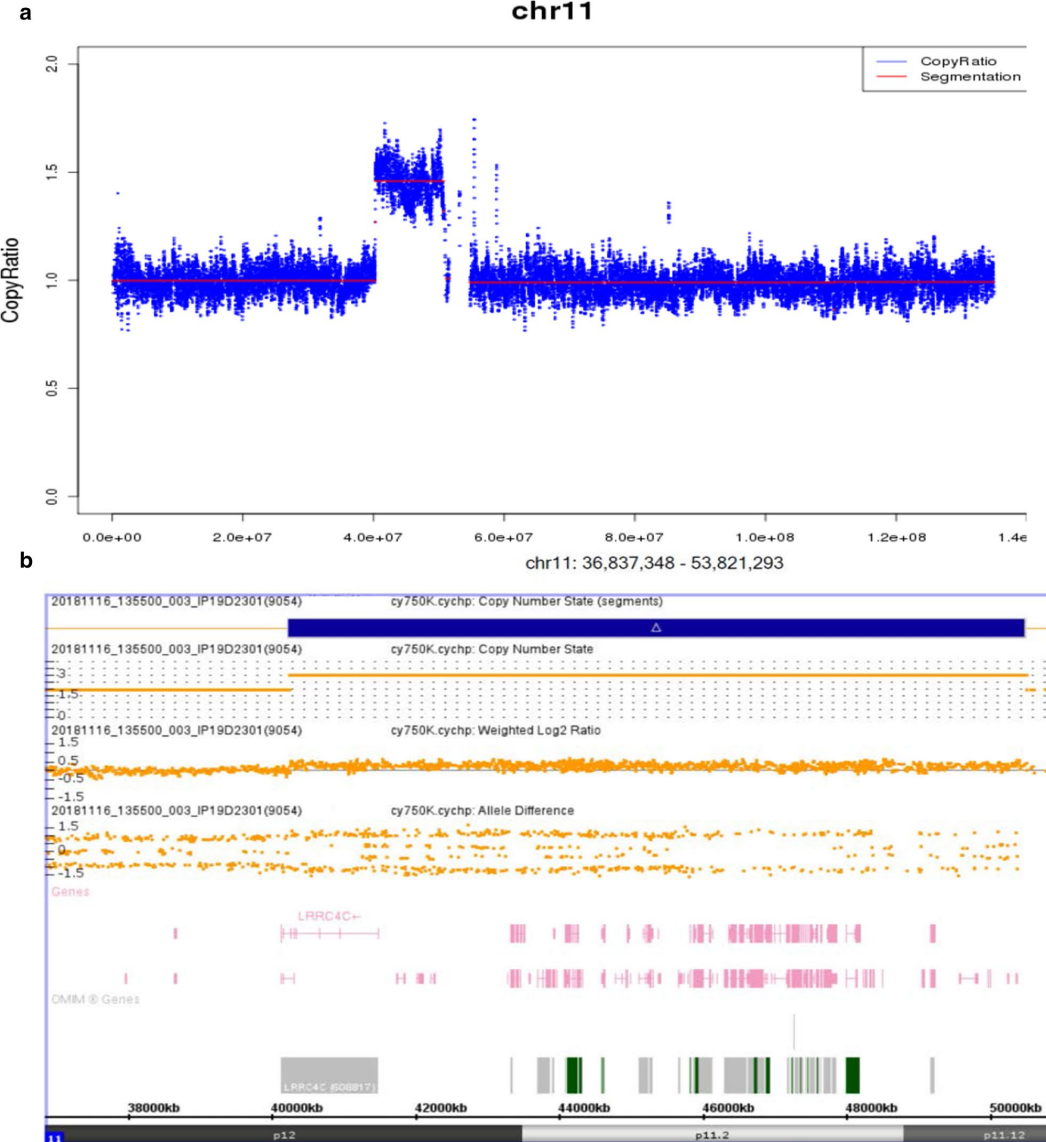
Molecular details. **a** A next-generation sequencing profile of the 10.53-Mb interstitial duplication at 11p11.12p12 in II4. **b** CMA profile of the 10.26-Mb duplication at 11p11.12p12 in II4, the same as in III1

We performed a karyotype analysis (G-banding-550 bands), and the result was 46, XX, dup(11)(p11.12p12) (Fig. 1b). In order to further clarify the location of the chromosome 11 duplications, CMA and next-generation sequencing analysis was performed. The results of sequencing analysis revealed a copy number increase of approximately 10.53 Mb in chromosome bands 11p11.12-p12 ranging from nucleotides 40231033_50762504 (Fig. 2a), a copy number loss of approximately 292.18 kb in chromosome bands 2q11.2, and a copy number increase of approximately 104.94 kb in chromosome bands 16p11.2 (Table 1). Microduplication of 16p11.2 was detected in 1 of 10,176 foetuses, with a normal phenotype based on follow-up, whereas the 11p11.12p12 duplication and 2q11.2 microdeletion were not found among these 10,176 foetuses (Table 1). In addition, microdeletion of 2q11.2 and microduplication of 16p11.2 involve no pathogenic genes, with full coverage of polymorphisms in the Database of Genetic Variants (DGV). The 11p11.12p12 duplication was confirmed by a third experiment involving CMA (Fig. 2b). We also collected peripheral blood from the patients' parents and performed karyotype analysis to determine the source of the 11p11.12p12 duplication. The chromosome karyotypes of the parents were normal (Table 1).

**Table 1:** Results of cytogenetic and molecular cytogenetic analysis in the family and 10,176 fetuses (2016–2019)

Family member	Karyotype	Next-generation sequencing	The SNP microarray analysis	Whole exome sequencing
I1	na	seq[GRCh37] (1–22) × 2, (XY) × 1	na	na
I2	na	seq[GRCh37] dup(4q12q13.1) (58.2–62.78 Mb) × 3, 4.58 Mb	na	na
I3	46, XY	seq[GRCh37] (1–22) × 2, (XY) × 1	na	na
I4	46, XX	seq[GRCh37] (1–22,X) × 2	na	na
II1	na	seq[GRCh37] dup(4q12q13.1) (58.18–62.76 Mb) × 3, 4.58 Mb	na	na
II2	na	seq[GRCh37] dup(4q12q13.1) (58.2–62.72 Mb) × 3, 4.52 Mb	na	na
II3	46, XY	seq[GRCh37] dup(4q12q13.1) (58.18–62.76 Mb) × 3, 4.58 Mb	na	arr[GRCh37] 4q12q13.1 (58193591_62730657) × 3, 4.54 Mb
II4 (proband)	46, XX, dup(11) (p11.12p12)	seq[GRCh37] del(2q11.2) (97727692_98019869) × 1, 292.18 Kb, dup(11p11.12p12) (40231033_50762504) × 3, 10.53 Mb, dup(16p11.2) (28 601618_28706557) × 3, 104.94 Kb	arr[GRCh37] 11p12p11.12(40242898_50501403) × 3, 10.26 Mb	arr[GRCh37] 11p12p11.12(40242898_50589224) × 3, 10.35 Mb
II5	na	seq (1–22,X) × 2	na	na
II6/II7	na	na	na	na
III1 (older son)	46, XY, dup(11) (p11.12p12)mat	seq[GRCh37] dup(4q12q13.1) (58.18 -62.76 Mb) × 3, 4.58 Mb, dup(11p11.12p12) (40.24–50.62 Mb) × 3, 10.38 Mb	arr[GRCh37] 4q12q13.1(58193591_62730657) × 3, 4.54 Mb, 11p12p11.12 (40242898_50589224) × 3, 10.35 Mb	arr[GRCh37] 4q12q13.1(58193591_62730657) × 3, 4.54 Mb 11p12p11.12(40242898_50589224) × 3, 10.35 Mb
III2	46, XY	seq[GRCh37] del(2q11.2) (97716795_98019869) × 1, 303.07 Kb dup(4q12q13.1) (58152873_62734056) × 3, 4.58 Mb, del(17p11.2) (21359750_21507889) × 1, 148.14 Kb, dup(22q11.21) (18655585_18894169) × 3, 238.58 Kb	na	na
10,176 fetuses	–	seq[GRCh37] dup 4q12q13.1(58.24–62.8 Mb) × 3, 4.56Mb^a^ seg[GRCh37] dup(16p11.2)(28.32–29.66 Mb) × 3, 1.34Mb^b^	na	na

na = not available. a = 1 identified case out of 10,176 foetuses was inherited from a phenotypically normal mother with a low weight and patent ductus arteriosus; this case was followed up after birth. b = 1 identified case out of 10,176 foetuses had a normal phenotype; this case was followed up after birth. A total of 10,176 foetal cases were identified by amniotic fluid microdeletion microduplication analysis, of which 2906 cases were detected by CMA and 7270 cases were detected by next-generation sequencing from 2016–2019

At 36 years, the proband’s physical examination in the neurology department showed resting tremor in both hands, which was obvious in the right hand. She had experienced confusion twice in 15 years. The electroencephalogram (EEG) results revealed moderate abnormalities, a small number of spikes as low as 110 microvolts and sharp slow waves were scattered, with the temporal region as the focus. Therefore, she was suspected to have seizures. Chest computed tomography (CT) displayed two cumulative changes in the lungs. Magnetic resonance imaging (MRI) of the brain showed abnormal signals of the centrum semiovale on both sides and a diffuse corona radiata, which suggested the possibility of demyelinating lesions. The analyses of lymphocyte subsets and immunoglobulin levels were normal.

III1 was the older son of the proband (Fig. 1e). His clinical phenotype included mild ID, suspected seizures, short stature, and CFA. He walked at 15 months. His first words were at 2 years. At 9 years, his neuropsychiatric evaluation revealed mild cognitive delay (IQ of 67 using the WISC-IV), speech delay and learning difficulties. His computational ability was poor; he could calculate one plus one but could not correctly calculate two plus two. He could write only approximately 100 simple words and could put fewer than three words together. In terms of self-care ability, he could neither correctly wear complicated clothes nor distinguish the left from the right shoe. Routine blood tests showed that red blood cell counts and haemoglobin content were slightly lower than normal. The results of trace element analysis and biochemical tests were normal. At 10 years, he could correctly distinguish the left from the right shoe but could still not distinguish between his left and right hands.

At the age of 13.5 years, he was 141.5 cm (− 2.83 SDs). His bone age was also 13.5 years according to left wrist X-ray (Fig. 1c). His testicular volume was approximately 12 ~ 15 ml (normal). His pubic hair was in stage PH1 (normal), and his growth hormone value was also normal at 14.1 ng/ml (reference value < 5.0 ng/ml). These data suggest that his developmental indicators were typical for his age of 13.5 years, but his height is significantly lower than the height of 13.5 y. Though his computing power and language skills had improved, he could still not speak in sentences of more than 5 words, and he could still not distinguish his left and right hands. He could correctly add 2 plus 2 or 4 plus 4 by memorization but still could not correctly add 1 plus 3.

At 14.6 years, physical examination in the neurology department revealed appendicular hypotonia and abnormal gait. Approximately a year ago, he had fallen asleep and could not be awakened for an hour. He was given an EEG examination, which displayed mild to moderate abnormalities, and small numbers of spiking waves as low as 90 microvolts were seen in the left posterior temporal and occipital regions. Thus, he was suspected seizures. Medical examinations, including echocardiographic examination, renal and urinary tract ultrasound, lymphocyte subset and immunoglobulin level analysis, chest CT, and MRI of the brain, were normal.

The karyotype analysis result of III1 was 46, XY, dup (11)(p11.12p12) mat. Molecular cytogenetic analysis of III1 revealed two duplications with a copy number increase in chromosome bands 11p11.12p12 (10.38 Mb) inherited from his mother and a copy number increase in chromosome bands 4q12q13.1 (4.58 Mb) inherited from his father. Moreover, 4q12q13.1 microduplication was found in one of 10,176 foetuses, with a low weight and patent ductus arteriosus; based on follow-up, the microduplication was inherited from a normal-phenotype mother. It was also found in 5 normal-phenotype members in the family (Table 1). To exclude abnormalities in other genes outside the 11p11.12p12 interval causing the phenotype of this family, we performed WES in II3, II4, and III1. No other gene anomalies were found (Table 1).

III2 was the younger son of the proband. During the second trimester, amniotic fluid karyotype analysis and next-generation sequencing analysis were performed. The results showed two microduplications and two microdeletions (Table 1). Del (2q11.2) and dup (4q12q13.1) were inherited from his mother and father, respectively. Del (17p11.2) and dup (22q11.21) were de novo copy number variations (CNVs), which were not found among 10,176 foetuses yet. Not all of the CNVs involving pathogenic genes were found in OMIM but were fully covered by GDV. No cases with phenotypes in the same interval of del (17p11.2) and dup (22q11.21) were found in the Database of Chromosomal Imbalance and Phenotype in Humans Using Ensembl Resources (DECIPHER). His karyotype was also normal. The results indicated that the foetus had no pathogenic chromosomal abnormalities, and the pregnancy continued to deliver. He began to learn to walk and vaguely said the word "dad" at 1 year old. He exhibited no significant difference compared with normal children in infancy and childhood. His height was 91 cm (− 1.53 SDs), and the difference between the younger son and the mean height of the family was -0.24 SDs. No significant differences in chromosome karyotype, developmental delay or ID were observed for the other members of the family.

## Discussion

With the exception of the 11p11.12p12 duplication, none of the microdeletions or microduplications in the pedigree contain the pathogenic genes included in OMIM and are fully covered by DGV. These microdeletions and microduplications were also observed in members of the family or other foetuses with a normal phenotype, although the 4q12q13.1 microduplication segments were longer than 4 Mb. A total of 11p11.12p12 segments containing 100 genes were detected only in the patient and her older son with the clinical phenotype. These findings suggest that the clinical phenotype of this family is caused by duplication of the chromosome 11 region. The 5-year follow-up data after the birth of the child also showed that the 11p11.12p12 duplication was the cause of the disease in this family, and our accurate genetic analysis of prenatal diagnosis provided the correct genetic counselling for the child.

**Fig. 3:**
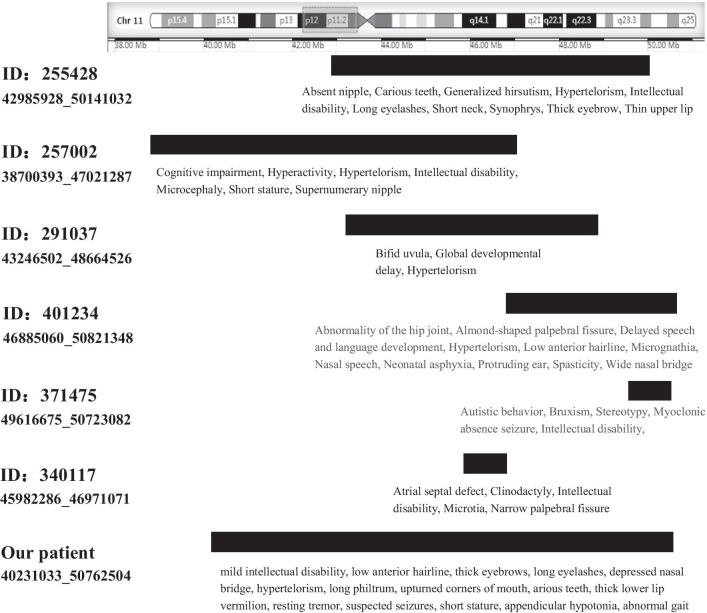
Chromosomal duplication or microduplication cases (> 1 Mb) in DECIPHER and our patient

Potocki–Shaffer syndrome (OMIM 601224) is a rare contiguous gene deletion syndrome due to haploinsufficiency of genes in chromosomal region 11p11.2p12 (chr11:43421550_48821552, GRCh37/hg19), similar to the segments duplicated in our patient (chr11:40231033_50762504Mb, GRCh37/hg19). Although the duplication interval (10 Mb) of our case was significantly larger than the PSS interval (5.4 Mb), there was no difference in the phenotypic genes they contained. PSS patients are very rare, with no more than 50 cases described in the literature. To determine the correlation between the genes contained in this chromosomal region and clinical phenotypes, we searched the clinical phenotypes of all PSS patients in an attempt to identify common characteristics. This syndrome is characterized by CFA, developmental delay, multiple exostoses, biparietal foramina, and ID [3–17]. PSS occasionally manifests as epilepsy, hypotonia and central nervous system anomalies [5–7, 9, 11–13, 17]. Craniofacial abnormalities include brachycephaly [8–10, 17], microcephaly [10–12, 17], bilateral parietal foramina [5, 6, 12, 15, 17], broad forehead [8, 10], high forehead [5, 7, 9, 13], laterally sparse eyebrows [7, 12, 15, 17], upslanting/downslanting palpebral fissures [5, 7, 10], bilateral epicanthal folds [6, 8, 10, 13, 14, 17], left ptosis [12, 14], esotropia [5, 6], hypertelorism [10, 13, 16], hypotelorism [10], mid-facial hypoplasia [11, 15], narrowed nasal bridge [10, 14, 15, 17], depressed nasal bridge [9, 10], broad nasal bridge [8, 16], short philtrum [6, 7, 14, 17], thin lips [7, 8, 10, 13], downturned mouth angle [5–7], dysplastic low-set ears [5, 10, 13], and mild micrognathia [6, 8, 10]. Most of the overlapping phenotypes appear in more than 3 studies and are most likely caused by 11p11.2p12 deletions. We also searched cases of chromosomal duplications or microduplications in the same chromosomal region, which were found only in the DECIPHER (Table 2). Most of the characteristics of our patient are consistent with those described in the DECIPHER database (Fig. 3). The most common clinical features of chromosome duplications in the 11p11.12p12 region are hypertelorism, ID, and thick eyebrows, short stature, followed by long eyelashes, EEG abnormalities, speech and expressive language delay, and cognitive impairment. The minimal clinical feature was ID, and the chromosome position was concentrated between 45427775 and 46949520 (GRCh38), a region that contains the *SLC35C1, MAPK8IP1, PEX16, CREB3L1, ZNF408, F2,* and *LRP4* genes. The critical region involved in PSS was also 11p11.2, spanning 2.1 Mb between D11S1393 to D11S1385/D11S1319 (44.6–46.7 Mb), and the gene related to ID may be located between 45.6 and 46.7 Mb [17]. Overall, there are common and distinct features between 11p11.2p12 deletion and duplication. The common clinical features include ID, hypertelorism, Micrognathia, hypotonia, Global developmental delay, and seizures. The distinct clinical features include long philtrum, low anterior hairline, thick eyebrows, upturned corners of mouth, and thick lower-lip vermilion (Table 2). The similarities and differences suggest that gene-dosage effects are not all linearly superimposed, especially in the development of the nervous system. The normal range of gene-dosage effects seems to be very narrow, similar to the equivalent zone of antigen–antibody reaction, and more or less doses will not be acceptable.

**Table 2:** Clinical features of 11p11.12p12 duplication and PSS

	The *proband*	The old son	ID: 257002	ID: 255428	ID: 291037	ID: 401234	ID: 371475	ID: 250532	ID: 412053	ID: 249534
Minimal coordinates, bp (GRCh38 or hg19)	40,231,033	40,242,898	38,678,843	42,964,378	43,224,952	46,885,060	49,616,675	48,088,490	47,266,966	49,811,971
Maxima coordinates, bp (GRCh38 or hg19)	50,762,504	50,589,224	46,999,736	50,181,861	48,642,974	50,821,348	50,723,082	48,870,325	47,439,621	50,421,230
Size	10.5 Mb	10.3 Mb	8.32 Mb	7.22 Mb	5.42 Mb	3.94 Mb	1.11 Mb	781.84 Kb	172.66 Kb	609.26 Kb
Inheritance/genotype	De novo Heterozygou	Maternally inherited Heterozygou	Unknown Heterozygous	De novo Heterozygous	De novo Heterozygous	De novo Heterozygous	Paternally inherited Heterozygous	Unknown Heterozygous	Maternally inherited Heterozygous	Unknown Heterozygous
Pathogenicity/contribution			–	–	Likely pathogenic Partial	Likely pathogenic	Likely benign None	–	Uncertain	–
*Abnormality of head or neck*										
Microcephaly	−	−	+	−	−	−	−	−	−	−
Brachycephaly	−	−	−	−	−	−	−	−	−	−
High forehead	−	−	−	−	−	−	−	−	−	−
Broad forehead	−	−	−	−	−	−	−	−	−	−
Low anterior hairline	+	+	−	−	−	+	−		−	−
Synophrys	−	+	−	+	−	−	−	+	−	−
Thick eyebrows	−	+	−	+	−	−	−	+	−	−
Sparse eyebrows	−	−	−	−	−	−	−	−	−	−
Long eyelashes	−	+	−	+	−	−	−	−	−	−
Hypertelorism	+	+	+	+	+	−	−	−	−	−
Hypotelorism	−	−	−	−	−	−	−	−	−	−
Narrow palpebral fissure	−	−	−	−	−	−	−	−	−	−
Upslanting/downslanting palpebral fissures	−	−	−	−	−	−	−	−	−	−
Almond-shaped palpebral fissure	−	−	−	−	−	+	−	−	−	−
Left ptosis	−	−	−	−	−	−	−	−	−	−
Epicanthus/epicanthal folds	−	−	−	−	−	−	−	−	−	−
Esotropia	−	−	−	−	−	−	−	−	−	−
Ear anomalies	−	−	−	−	−	+	−	−	−	−
Mid-facial hypoplasia	−	−	−	−	−	−	−	−	−	−
Narrowed nasal bridge	−	−	−	−	−	−	−	−	−	−
Depressed nasal bridge	+	−	−	−	−	−	−	−	−	−
Broad nasal bridge	−	−	−	−	−	+	−	−	−	−
Long philtrum	+	+	−	−	−	−	−	−	−	−
Short philtrum	−	−	−	−	−	−	−	−	−	−
High palate	−	−	−	−	−	−	−	+	−	−
Bifid uvula	−	−	−	−	+	−	−	−	−	−
Upturned corners of mouth	±	+	−	−	−	−	−	−	−	−
Downturned mouth angle	−	−	−	−	−	−	−	−	−	−
Thick lower lip vermilion	±	+	−	−	−	−	−	−	−	−
Thin upper lip vermilion	−	−	−	+	−	−	−	−	−	−
Thin lips	−	−	−	−	−	−	−	−	−	−
Carious teeth	+	+	−	+	−	−	−	−	−	−
Micrognathia	−	−	−	−	−	+	−	−	−	+
Short neck	−	−	−	+	−	−	−	−	−	−
Triangular face	−	−	−	−	−	−	−	−	−	−
Abnormality of the nervous system										
Intellectual disability	+	+	+	+	−	−	+	+	−	−
Global developmental delay	−	−	−	−	+	−	−	−	−	−
Hyperactivity	−	−	+	−	−	−	−	−	+	−
Cognitive impairment	−	−	+	−	−	−	−	−	−	−
Speech delay	+	+	−	−	−	+	−	−	+	−
Autism	−	−	−	−	−	−	+	+	+	−
Resting tremor	+	−	−	−	−	−	−	−	−	−
Gait disturbance	−	+	−	−	−	−	−	−	+	−
Epilepsy/seizures	?	?	−	−	−	−	+	−	−	−
Stereotypy	−	−	−	−	−	−	+	−	+	−
*Abnormality of the skeletal/muscular system*										
Biparietal foramina	−	−	−	−	−	−	−	−	−	−
Multiple exostoses	−	−	−	−	−	−	−	−	−	−
Clinodactyly	−	−	−	−	−	−	−	−	−	−
Abnormality of the hip joint	−	−	−	−	−	+	−	−	−	−
Scoliosis	−	−	−	−	−	−	−	−	−	+
Abnormality of digit	−	−	−	−	−	−	−	+	−	+
Spasticity	−	−	−	−	−	+	−	−	−	−
Appendicular hypotonia	−	+	−	−	−	−	−	−	−	+
*Other*										
Short stature	+	+	+	−	−	−	−	−	−	+
Generalized hirsutism	−	−	−	+	−	−	−	−	−	−
Breast anomalies	−	−	+	+	−	−	−	−	−	−
Atrial septal defect	−	−	−	−	−	−	−	−	−	−
EEG abnormality	+	+	−	−	−	−	−	−	−	−
Constipation	−	−	−	−	−	−	−	−	+	−
Neonatal asphyxia	−	−	−	−	−	+	−	−	−	−
Micropenis	−	−	−	−	−	−	−	−	−	−

	ID: 282366	ID: 285181	ID: 290203	ID: 289550	ID: 290135	ID: 331490	ID: 288560	ID: 307922	ID: 340117	PSS^*^
Minimal coordinates, bp (GRCh38 or hg19)	48,051,524	48,099,784	50,013,207	49,784,894	47,641,574	45,427,775	46,455,773	46,420,985	45,960,735	34,006,368 McCool C et al. [15]
Maxima coordinates, bp (GRCh38 or hg19)	48,445,343	48,643,003	50,821,348	49,976,474	47,743,809	46,158,994	46,791,939	46,787,571	46,949,520	51,387,923 Ferrarini A et al. [16]
Size	393.82 Kb	543.22 Kb	808.14 Kb	191.58 Kb	102.24 Kb	731.22 Kb	336.17 Kb	366.59 Kb	988.79 Kb	137 Kb − 11 Mb
Inheritance/genotype	Unknown Heterozygous	Maternally inherited Heterozygous	Maternally inherited Heterozygous	Maternally inherited Heterozygous	Paternally inherited Heterozygous	Paternally inherited Heterozygous	Maternally inherited Heterozygous	Paternally inherited Heterozygous	Unknown Heterozygous	–
Pathogenicity/contribution	–	Likely benign	Likely benign	Likely benign	Uncertain	Likely pathogenic	Likely benign	Likely benign Uncertain	Uncertain	–
*Abnormality of head or neck*										
Microcephaly	−	−	−	−	−	−	−	−	−	+
Brachycephaly	−	−	−	−	−	−	−	−	−	+
High forehead	−	−	−	−	−	−	−	−	−	+
Broad forehead	−	−	−	−	−	−	−	−	−	+
Low anterior hairline	−	−	−	−	−	−	−	−	−	−
Synophrys	−	−	−	−	−	−	−	−	−	−
Thick eyebrows	−	−	−	−	−	−	−	−	−	−
Sparse eyebrows	−	−	−	−	−	−	−	−	−	+
Long eyelashes	−	+	−	−	−	−	−	−	−	−
Hypertelorism	−	−	−	−	−	−	−	−	−	+
Hypotelorism	−	−	−	−	−	−	−	−	−	+
Narrow palpebral fissure	−	−	−	−	−	−	−	−	+	−
Upslanting/downslanting palpebral fissures	+	−	−	−	−	−	−	−	−	+
Almond-shaped palpebral fissure	−	−	−	−	−	−	−	−	−	−
Left ptosis	−	−	−	−	−	−	−	−	−	+
Epicanthus/epicanthal folds	−	−	−	−	−	−	−	+	−	+
Esotropia	−	−	−	−	−	−	−	−	−	+
Ear anomalies	−	−	−	−	−	−	−	−	+	+
Mid-facial hypoplasia	−	−	−	−	−	−	−	−	−	+
Narrowed nasal bridge	−	−	−	−	−	−	−	−	−	+
Depressed nasal bridge	−	−	−	−	−	−	−	−	−	+
Broad nasal bridge	−	−	−	−	−	−	−	−	−	+
Long philtrum	−	−	−	−	−	−	−	−	−	−
Short philtrum	−	−	−	−	−	−	−	−	−	+
High palate	−	−	−	−	−	−	−	+	−	−
Bifid uvula	−	−	−	−	−	−	−	−	−	−
Upturned corners of mouth	−	−	−	−	−	−	−	−	−	−
Downturned mouth angle	−	−	−	−	−	−	−	−	−	+
Thick lower lip vermilion	−	−	−	−	−	−	−	−	−	−
Thin upper lip vermilion	−	−	−	−	−	−	−	−	−	−
Thin lips	−	−	−	−	−	−	−	−	−	+
Carious teeth	−	−	−	−	−	−	−	−	−	−
Micrognathia	−	−	−	−	−	−	−	−	−	+
Short neck	−	−	−	−	−	−	−	−	−	−
Triangular face	−	+	−	−	−	−	−	−	−	+
Abnormality of the nervous system										
Intellectual disability	−	−	−	+	+	+	+	−	+	+
Global developmental delay	−	+	+	−	−	−	−	−	−	+
Hyperactivity	−	−	−	−	−	−	−	−	−	−
Cognitive impairment	−	−	−	−	−	−	−	−	−	−
Speech delay	−	−	+	−	−	−	−	+	−	+
Autism	−	−	−	+	−	−	−	−	−	−
Resting tremor	−	−	−	−	−	−	−	−	−	−
Gait disturbance	−	−	−	−	−	−	−	−	−	−
Epilepsy/seizures	−	−	−	−	−	−	−	−	−	+
Stereotypy	−	−	−	−	−	−	−	−	−	−
*Abnormality of the skeletal/muscular system*										
Biparietal foramina	−	−	−	−	−	−	−	−	−	+
Multiple exostoses	−	−	−	−	−	−	−	−	−	+
Clinodactyly	−	−	−	−	−	−	−	−	+	−
Abnormality of the hip joint	−	−	−	−	−	−	−	−	−	−
Scoliosis	−	−	−	−	−	−	−	−	−	−
Abnormality of digit	+	+	−	−	−	−	−	−	−	−
Spasticity	−	−	−	−	−	−	−	−	−	−
Appendicular hypotonia	+	−	−	−	−	−	−	−	−	+
*Other*										
Short stature	−	−	−	−	−	−	−	−	−	−
Generalized hirsutism	−	−	−	−	−	−	−	−	−	−
Breast anomalies	−	−	−	−	−	−	−	−	−	−
Atrial septal defect	−	−	−	−	−	−	−	−	+	−
EEG abnormality	−	−	−	−	−	−	−	+	−	−
Constipation	−	−	−	−	−	−	−	+	−	−
Neonatal asphyxia	−	−	−	−	−	−	−	−	−	−
Micropenis	−	−	−	−	−	−	−	−	−	+

‘−’ = the absence of the corresponding phenotype. ‘?’ = suspected seizures. ‘^*^’ = The clinical features of PSS in literature from reference [3–17]

We know that 11p11.12p12 encompasses approximately 18 morbid genes, including *EXT2* (OMIM*608210), *ALX4* (OMIM*605420), *CD82* (OMIM*600623), *SLC35C1* (OMIM*605881), *MAPK8IP1* (OMIM*604641), *MYBPC3* (OMIM*600958), *PHF21A* (OMIM*608325), *LRP4* (OMIM*604270), *CREB3L1* (OMIM*616215), *ZNF408* (OMIM*616454), *F2* (OMIM*176930), *DDB2* (OMIM*600811), *ACP2* (OMIM*171650), *SLC39A13* (OMIM*608735), *RAPSN* (OMIM*601592), *PEX16* (OMIM*603360), *NDUFS3* (OMIM*256000), and *PTPRJ* (OMIM*600925). Which genes within the interval contribute to 11p11.12p12 duplication syndrome and even PSS remains to be determined. *EXT2* is an important pathogenic gene in PSS because its mutation mainly causes multiple exostoses. *ALX4* is expressed in various organs and plays an essential role in the development of the skull, limbs, and skin [18]. *ALX4* mutations result in functional haploinsufficiency, such as the development of frontonasal dysplasia 2, impaired interfollicular epidermal differentiation and perturbed regular hair follicle differentiation [19, 20]. This gene may be responsible for some of our patient’s craniofacial abnormalities. The *SLC35C1* gene encodes a GDP-fucose transmembrane transporter (FucT1) located in the Golgi apparatus [21]. It is abundantly expressed in the brain, gastrointestinal tract, female tissues, male tissues, kidney and urinary bladder, bone marrow, and lymphoid tissues. Furthermore, RNA tissue-specific expression is enhanced in the liver in HPA (https://www.proteinatlas.org). SLC35C1 is a negative regulator of Wnt signalling in colon cancer. In fact, overexpressing SLC35C1 inhibits the canonical Wnt pathway [22]. *SLC35C1* heterozygous mutations may cause partial in vivo defects in fucosylation [23]. The *PHF21A* gene encodes BHC80, a component of the BRAF35/histone deacetylase complex (BHC), which mediates repression of neuron-specific genes through the cis-regulatory element known as repressor element-1 (RE1) or neural restrictive silencer (NRS). Based on RT-PCR, the highest level of tissue-specific expression occurs in the brain [24]. PHF21A is the likely cause of ID and craniofacial abnormalities in Potocki–Shaffer Syndrome [25]. *MAPK8IP1*, also called Islet-brain-1 (*IB1*) or JNK-interacting protein-1 (*JIP1*), is abundantly expressed in the pancreas, brain, testis, and prostate and, to a lesser extent, in the heart, ovary, and small intestine [26]. HPA exhibits tissue-specific expression in the brain, with the strongest expression in the cerebral cortex. MAPK8IP1/JIP1 is a critical regulator of autophagosome transport in neurons, which ensures the fidelity of retrograde autophagosome transport in the axon and is highly sensitive to defects in autophagy [27]. JIP1 also plays an important role in neurons as a regulator of kinesin-1-dependent transport [28]. *RAPSN*, which is considered to be related to brain/cognition in DDG2P, shows low expression in HPA. Therefore, overexpression of this gene would not cause ID in this syndrome. *ALX4*, *SLC35C1*, *PHF21A*, and *MAPK8IP1* may be the genes most likely to have a gene-dosage effect in 11p11.12p12 duplication. Further work is required to fully elucidate the mechanisms leading to 11p11.12p12 duplication. In ClinGen, there is no evidence for triplosensitive phenotypes for any of the above genes and only evidence for haploinsufficiency phenotypes. This study suggests that there are triplosensitive effect genes responsible for 11p11.12p12-related syndrome.

We present two patients in a family with de novo 11p11.12p12 duplication and provide the results of five years of clinical follow-up. The duplication was identified and evaluated using a multidisciplinary protocol involving assessment by a geneticist, stomatologist, neurologist, psychiatrist, and paediatrician. The clinical features include mild ID, short stature, craniofacial anomalies, suspected seizures, resting tremor, appendicular hypotonia, and abnormal gait. The identification of clinical phenotypes will facilitate genetic counselling, especially prenatal genetic counselling.

## Conclusions

We report the first 11p11.12p12 duplication in a family with ID and craniofacial abnormalities. The findings will facilitate the clinical diagnosis and genetic counselling of patients with 11p11.12p12 duplication in the future. Karyotype analysis and molecular cytogenetics were helpful to identify the genetic aetiology of the patients in the family and potential candidate genes with triplosensitive effects involved in 11p11.12p12 duplication.

## Data Availability

The datasets generated and/or analysed during the current study are available in the NCBI Sequence Read Archive (SRA) repository at https://www.ncbi.nlm.nih.gov/sra/ PRJNA713823, and the Gene Expression Omnibus (GEO) repository at https://www.ncbi.nlm.nih.gov/geo/query/acc.cgi?acc=GSE169469.

## Abbreviations

PSS: Potocki–Shaffer syndrome
EEG: Electroencephalogram
MRI: Magnetic resonance imaging
CMA: Chromosome microarray analysis
WES: Whole-exome sequencing
SNVs: Single nucleotide variations
ID: Intellectual disability
CFA: Craniofacial anomalies
ISCN: International System for Human Cytogenomic Nomenclature
PCR: Polymerase chain reaction
ChAS: Chromosome Analysis Suite
QC: Quality control
HGMD: Human Gene Mutation Database
OMIM: Online Mendelian Inheritance in Man
BWA: Burrows-Wheeler Aligner
DGV: Database of Genetic Variants
CT: Computed tomography
CNVs: Copy number variations
DECIPHER: Database of Chromosomal Imbalance and Phenotype in Humans using Ensembl Resources
FucT1: Fucose transmembrane transporter
BHC: BRAF35/histone deacetylase complex
RE1: Repressor element-1
NRS: Neural restrictive silencer
IB1: Islet-brain-1
JIP1: JNK-interacting protein-1
SRA: Sequence Read Archive
GEO: Gene Expression Omnibus
